# The Performance of a Survival Nomogram and Albumin–Bilirubin Grade as Prognostic Tools in Advanced Hepatocellular Carcinoma Treated with FOLFOX4

**DOI:** 10.3390/jpm14040403

**Published:** 2024-04-11

**Authors:** Jirapat Wonglhow, Patrapim Sunpaweravong, Chirawadee Sathitruangsak, Arunee Dechaphunkul

**Affiliations:** Division of Medical Oncology, Department of Internal Medicine, Faculty of Medicine, Prince of Songkla University, Songkhla 90110, Thailand; jirapat.jw@gmail.com (J.W.); spatrapi@medicine.psu.ac.th (P.S.); sjirawadee@gmail.com (C.S.)

**Keywords:** hepatocellular carcinoma, liver cancer, chemotherapy, prognostic tool, ALBI grade, survival nomogram

## Abstract

Background: The ability of the survival nomogram developed in the EACH study and albumin–bilirubin (ALBI) grade to predict the survival of advanced hepatocellular carcinoma (HCC) patients receiving oxaliplatin plus 5-fluorouracil/leucovorin (FOLFOX4) remains unvalidated. Here, we comprehensively evaluated these prognostic tools. Methods: The survival nomogram and ALBI grade of each patient were assessed, and the area under the receiver operating curve (AUROC) and Harrell’s C-index for the risk classification model were calculated. Results: Overall, 76 HCC patients who received FOLFOX4 between August 2017 and June 2023 were included. The survival nomogram classified patients into low-, intermediate-, and high-risk groups, with a median overall survival (OS) of 9.82, 10.64, and 3.70 months, respectively (*p* = 0.23). The AUROC was 0.621 and Harrell’s C-index was 0.589. However, the ALBI grade categorized all patients into grade 1, 2, and 3, with a median OS of 9.82, 6.83, and 1.58 months, respectively (*p* = 0.00024). The AUROC was 0.663 and Harrell’s C-index was 0.663. Conclusion: The ALBI grade can be a potential prognostic tool. However, the survival nomogram does not provide clear discrimination. Therefore, FOLFOX4 should be an option for patients with ALBI grade 1 who cannot receive immunotherapy or targeted therapy. Additional prospective studies with a larger cohort are warranted to validate the survival nomogram and ALBI grade as prognostic tools.

## 1. Introduction

Hepatocellular carcinoma (HCC), a predominant form of liver cancer, is the sixth most commonly diagnosed cancer and the third leading cause of cancer-related deaths worldwide in 2020 [1]. In Southeast Asian nations, including Thailand, HCC substantially contributes to cancer-related deaths in both men and women [1,2]. However, the overall prognosis of HCC remains poor worldwide, leading to almost similar incidence and mortality rates. The estimated global incidence of liver cancer may increase by approximately 30% in the year 2030, with a corresponding increase in mortality [3].

While routine screening has been implemented to detect early-stage HCC in high-risk groups, HCC still frequently presents as a locally advanced or metastatic disease at the time of diagnosis [4,5]. Treatments for advanced HCC mainly involve systemic therapies like targeted therapies and immunotherapies. These treatments are crucial for improving overall survival [6,7,8,9,10]. However, the effectiveness of chemotherapy remains uncertain [11]. Nevertheless, owing to affordability issues, the use of both immunotherapy and targeted therapy is limited in Thailand. Therefore, providing the best supportive care, with or without palliative chemotherapy, is considerably important for patients with a favorable performance status in Thailand.

When considering chemotherapy, the EACH study [12,13], conducted in Asian countries, provides substantial evidence for using the oxaliplatin plus 5-fluorouracil/leucovorin (FOLFOX4) regimen for patients with advanced HCC. Furthermore, a cost-effectiveness analysis comparing FOLFOX4 and sorafenib in China has revealed that FOLFOX4 provides more value [14]. Therefore, studies on the potential advantages of FOLFOX4 for Thai patients with advanced HCC are warranted.

At present, widely used prognostic tools to indicate the potential benefits of chemotherapy for individuals with advanced HCC are lacking. A survival nomogram introduced by Qin et al. (2017) is the only existing tool; this nomogram uses individual patient profiles from those receiving FOLFOX4 treatment for advanced HCC [15]. Six variables have been integrated into this prognostic model (Supplementary Figure S1): age, maximum tumor diameter, lymph node status, aspartate aminotransferase (AST), total bilirubin (TB), and alpha-fetoprotein (AFP). The calculated score can classify patients with HCC into three distinct risk categories: low-, intermediate-, and high-risk groups, with 6-month survival probabilities of >70%, 30–70%, and <30%, respectively. In addition, compared with other recognized staging systems such as the Barcelona Clinic Liver Cancer (BCLC), American Joint Committee on Cancer, Chinese University Prognostic Index, and Groupe d’Étude et de Traitement du Carcinome Hépatocellulaire, this survival nomogram can precisely predict patient survival [16,17,18].

The albumin–bilirubin (ALBI) grade has emerged as an alternative and objective tool to assess liver functional reserve in individuals with HCC, categorizing them into three grades. The ALBI grade can differentiate between various patient subsets with distinct prognoses across various BCLC stages and Child–Turcotte–Pugh (CTP) classes; therefore, it is an attractive clinical prognostic indicator. There is a correlation between the ALBI grade and survival rates in patients who receive various multikinase inhibitors, including sorafenib, lenvatinib, cabozantinib, and regorafenib, and immune checkpoint inhibitor therapy [19].

Although internal validation has revealed that the survival nomogram exhibits robust performance in the Chinese population, its applicability to individuals with different characteristics and backgrounds warrants additional investigation. Furthermore, there is no confirmation regarding the validity of the ALBI grading system in individuals who have received chemotherapy. Therefore, here, we comprehensively evaluated and validated the prognostic tools the survival nomogram and the ALBI grade in patients with advanced HCC who received FOLFOX4.

## 2. Materials and Methods

### 2.1. Study Participants and Procedure

This retrospective, single-center study was conducted at Prince of Songkla University Hospital. It included 76 patients who underwent palliative chemotherapy using the FOLFOX4 regimen between August 2017 and June 2023. The treatment regimen was administered either as a first-line or later-line approach at the Medical Oncology Service. The inclusion criteria were as follows: (1) HCC diagnosis confirmed using typical imaging criteria or histological diagnosis in which typical imaging criteria included a hypervascular pattern with arterial enhancement and rapid washout during the portal venous or delayed phase; (2) patients with advanced disease (failed or refractory to local treatment, metastatic, and/or recurrent); (3) completion of at least one cycle of the FOLFOX4 regimen; and (4) patients aged ≥ 18 years or older. The exclusion criteria were as follows: patients without the information needed to assess the survival nomogram and ALBI grade, including age, maximum tumor diameter, lymph node status, AST, TB, AFP, and albumin.

Patient information was obtained from electronic medical records using the hospital information system (HIS) of Prince of Songkla University Hospital. Information on initial clinical characteristics, including age at diagnosis, sex, body weight, body mass index, Eastern Cooperative Oncology Group performance status, presence of cirrhosis, causes of cirrhosis, CTP score, BCLC stage, diameter of the largest tumor, number of liver tumor counts, extrahepatic metastasis status, and baseline laboratory data, was collected.

Abdominal radiologists confirmed HCC diagnosis and quantified tumor burden, encompassing parameters such as tumor size and number, and documented them in the HIS. This study was reviewed and approved by the Ethics Committee of the Research Center of the Faculty of Medicine, Prince of Songkla University (REC.66361141). Written informed consent was waived, owing to the retrospective nature of the study. To protect patient safety, all identifying information has been removed from this study.

The FOLFOX4 chemotherapy regimen was administered as follows: on day 1, oxaliplatin was infused at a dose of 85 mg/m^2^ for over 2 h, concurrently with leucovorin (LV) at 200 mg/m^2^. Thereafter, an intravenous bolus injection of 5-fluorouracil (5-FU) was administered at 400 mg/m^2^, followed by a 22 h infusion of 5-FU at 600 mg/m^2^, which was immediately initiated after the 5-FU bolus. LV and 5-FU were reintroduced on day 2 of the treatment cycle. The FOLFOX4 regimen was repeated at 2-week intervals and continued until disease progression, death, the onset of intolerable side effects, or upon the patient’s indication of preference. If the FOLFOX4 regimen was ineffective, subsequent therapeutic protocols were contemplated. The decision was based on the patient’s performance status, patient’s personal preference, or feasibility of alternate agents.

### 2.2. Measurement

The primary study objective was to determine the effectiveness of the proposed survival nomogram and the ALBI grade as prognostic tools for patients receiving the FOLFOX4 regimen for advanced HCC. The secondary objectives were evaluating the outcomes of the FOLFOX4 regimen in terms of OS, progression-free survival (PFS), and objective response rate (ORR). OS was defined as the time from initiating the FOLFOX4 regimen to death from any causes. PFS was defined as the duration from initiating the FOLFOX4 regimen to either radiologically identified tumor progression or death, whichever occurred first. Disease progression was defined, and the response rate was evaluated using the Response Evaluation Criteria for Solid Tumors 1.1. Abdominopelvic and/or chest computed tomography (CT) were performed to elucidate treatment responses every 2–3 months. Response rates were determined for all patients (intention-to-treat [ITT] analysis) as well as for those with assessable data. Supplementary Figures S1 and S2 illustrate the proposed survival nomogram and the formula used to calculate the ALBI grade and its interpretation, respectively.

### 2.3. Statistical Analysis

In terms of baseline characteristics, continuous variables were presented as median and interquartile range (IQR) or as mean and standard deviation where appropriate. In contrast, categorical variables were presented as frequencies and corresponding percentages. The proposed survival nomogram and the ALBI grade were assessed for each patient, who were categorized into three distinct risk groups, i.e., low-, intermediate-, and high-risk groups, and three grades, i.e., 1, 2, and 3, respectively. The Kaplan–Meier method was used to generate survival curves, which were compared using the log-rank test. Harrell’s concordance index (C-index) and the area under the receiver operating characteristic curve (AUROC) were used to determine the discriminatory capacity of the prognostic tools. R software version 3.3.2 (R Foundation, Vienna, Austria) was used to perform statistical analyses. All *p*-values were considered two-sided, and a *p*-value of 0.05 was considered statistically significant.

## 3. Results

### 3.1. Baseline Characteristics

In this study, 76 patients who underwent the FOLFOX4 regimen between August 2017 and June 2023 were included. These patients encompassed the entire cohort. The data collection period ended on 31 August 2023. Among the included patients, 56 (73.7%) received the FOLFOX4 regimen as the first-line treatment and were referred to the first-line cohort. On the other hand, the remaining 20 patients (26.3%) received the FOLFOX4 regimen as the second- to later-line treatment and were called the later-line cohort. Table 1 summarizes the baseline clinical characteristics and laboratory results of the patients.

The mean age at diagnosis was 56.5 years, and cirrhosis was the prevalent condition among most patients (90.8%). Most patients (73.7%) were classified as CTP class A, whereas the remaining were categorized as CTP class B. The average diameter of the largest primary tumor was 11.0 cm. Approximately 88.2% of the patients were diagnosed with BCLC stage C, and approximately 50% of the patients exhibited extrahepatic metastasis (56.6%) and portal vein involvement (52.6%). The most common metastatic site was the lungs (25.0%), followed by the lymph nodes (18.4%) and peritoneum (14.5%). In terms of baseline laboratory outcomes, the median TB, AST, and AFP levels were 1.2 mg/dL, 101.5 U/L, and 5630.5 ng/dL, respectively, whereas the mean serum albumin level was 3.5 g/dL.

The survival nomogram was used to calculate the prognostic score of each patient. As a result, the patients were categorized into three distinct risk groups: low-, intermediate-, and high-risk groups, comprising 22.4, 25.0, and 52.6% of the entire cohort, respectively (Supplementary Table S1). Furthermore, the ALBI grade of each patient was calculated and the patients were classified into the following three grades: 1, 2, and 3, comprising 19.7%, 67.1%, and 13.2% of the entire cohort, respectively (Supplementary Table S1).

### 3.2. Treatment Information

In the entire cohort, the median number of FOLFOX4 cycles was three, with two cycles for the first-line cohort and five for the later-line cohort. Supplementary Table S2 summarizes the FOLFOX4 regimen and subsequent treatment details. Approximately 47.4% of the patients started with a decreased oxaliplatin dose, and 8.6% of the patients with a decreased 5-FU dose. During the subsequent cycles, 75.9% had a decreased oxaliplatin dose, whereas 11.8% had a decreased 5-FU dose. Disease progression was the primary reason for treatment discontinuation. Among the patients who received the FOLFOX4 regimen and suffered from disease progression, only 15.8% received subsequent lines of systemic therapy, including doxorubicin (7.9%), and continued FOLFOX4 beyond progression (3.9%).

### 3.3. OS

The median follow-up duration was 4.9 months (IQR 2.4, 12.6). The median OS of the entire cohort was 5.32 months. Furthermore, the median OS of the first-line cohort was 4.98 months. In contrast, the median OS of the later-line cohort was 9.82 months (Supplementary Figure S3).

### 3.4. PFS

The median PFS of the entire cohort was 4.11 months. Furthermore, the median PFS of the first-line cohort was 3.70 months; in contrast, the median PFS of the later-line cohort was 4.73 months (Supplementary Figure S4).

### 3.5. ORR

Among the 76 patients who received the FOLFOX4 regimen within the entire cohort, comprehensive radiological evaluations were available for 52.6% of patients. The ITT analysis revealed that the ORR of the entire cohort was 11.8%. However, this rate increased to 22.5% when considering patients with evaluable radiological data, as elaborated in Table 2.

### 3.6. Effectiveness of the Proposed Survival Nomogram

**Figure 1:**
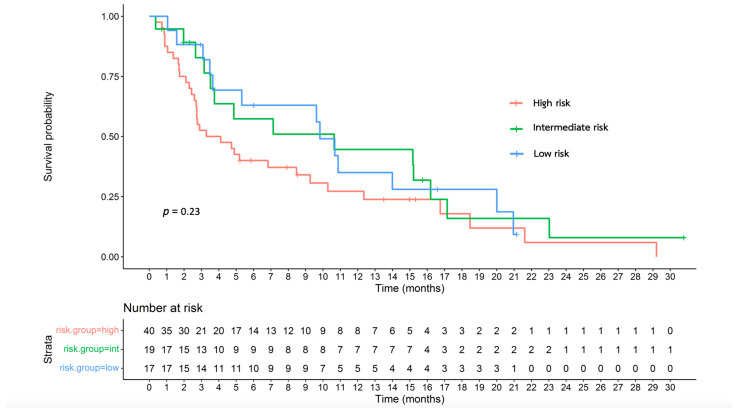
Overall survival of the risk groups of the entire cohort classified based on the proposed survival nomogram.

Table 3 summarizes the median OS of the different risk groups in each cohort. The survival nomogram of the entire patient cohort was used to divide the patients into risk categories. The median OS of the patients in the low-, intermediate-, and high-risk groups was 9.82, 10.64, and 3.70 months, respectively (Figure 1; *p* = 0.23). For the first-line cohort, the median OS of the patients in the low-, intermediate-, and high-risk groups was 5.32, 8.89, and 3.29 months, respectively (Supplementary Figure S5; *p* = 0.62).

**Figure 2:**
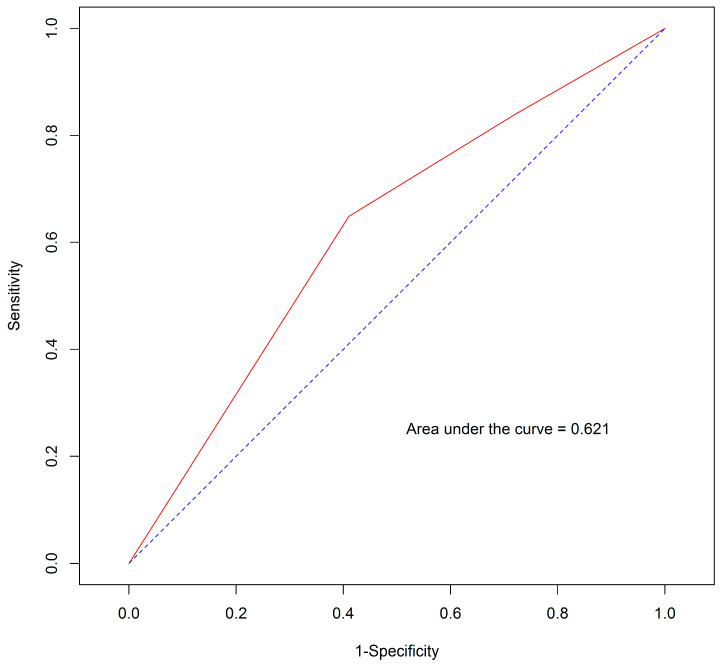
Area under the receiver operating characteristic curve (AUROC) of the proposed survival nomogram. The blue line represents the reference line of the receiver operating characteristic (ROC) curve with an AUROC of 0.5, indicating no discrimination ability (random effect). The red line represents the ROC curve of the proposed survival nomogram, showing an AUROC of 0.621.

The multivariate Cox proportional hazards regression model was used to assess nomogram variables to predict survival. Evidently, only TB exhibited a statistically significant effect (Supplementary Table S3). When the proposed survival nomogram was applied to our patient group, the AUROC was 0.621 (Figure 2). Furthermore, Harrell’s C-index value was 0.589.

### 3.7. Effectiveness of the ALBI Grade

**Figure 3:**
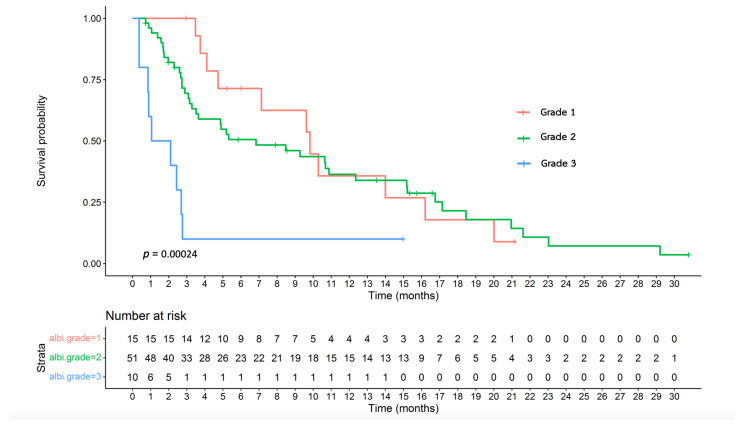
Overall survival based on the ALBI grade of the entire cohort.

Table 4 summarizes the median OS of the patients categorized based on the ALBI grade. Stratifying the entire cohort based on the ALBI grade revealed median OS values of 9.82, 6.83, and 1.58 months for ALBI grades 1, 2, and 3, respectively (Figure 3; *p* = 0.00024). Furthermore, in the first-line cohort, stratification based on the ALBI grade revealed that the patients with ALBI grade 1 achieved a median OS of 9.63 months, whereas those with ALBI grades 2 and 3 exhibited median OS durations of 5.26 and 1.05 months, respectively (Supplementary Figure S6; *p* = 0.0025).

**Figure 4:**
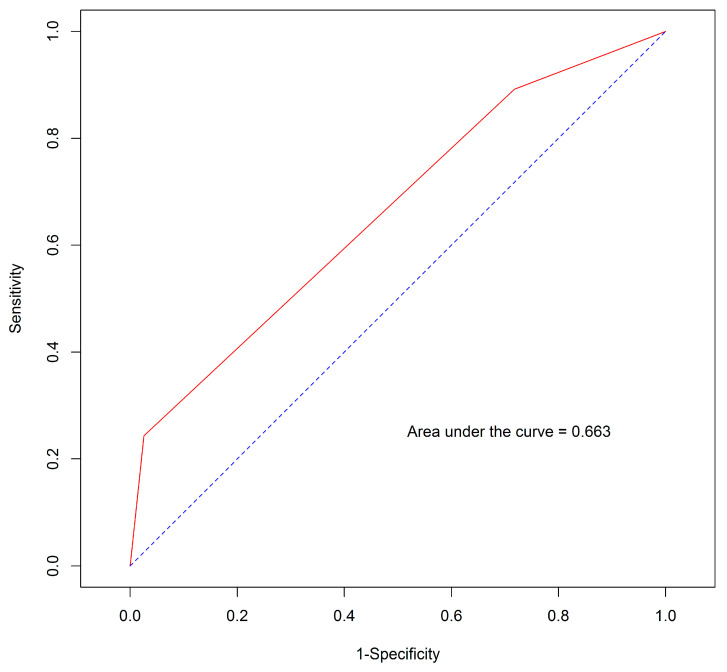
Area under the receiver operating characteristic curve (AUROC) of the ALBI grade. The blue line represents the reference line of the receiver operating characteristic (ROC) curve with an AUROC of 0.5, indicating no discrimination ability (random effect). The red line represents the ROC curve of the ALBI grade, showing an AUROC of 0.663.

The multivariate Cox proportional hazards regression model was used to evaluate the effect of ALBI grade variables on survival prediction. Only TB exhibited a statistically significant effect (Supplementary Table S4). The application of the ALBI grade to our patient group resulted in an AUROC value of 0.663 (Figure 4) and Harrell’s C-index of 0.663.

## 4. Discussion

In the present study, we evaluated the effectiveness of two specific prognostic tools by externally validating the survival nomogram and validating the ALBI grade for the first time in the chemotherapy settings and applied them to patients with advanced HCC who received the FOLFOX4 regimen.

We observed that the proposed survival nomogram was not fit for our patient population. In particular, whether considering the entire cohort or the subset receiving the first-line treatment, the calculated scores could not distinctly discriminate the survival outcomes of our patients based on risk groups. Nevertheless, we confirmed a notable trend wherein the high-risk group exhibited a tendency toward worse outcomes compared with the other risk groups.

The following factors may have contributed to the deviation of our results from the original data of the EACH study [12,15]:

1. Our study is limited by a relatively small sample size, i.e., 76 patients, in contrast to a larger cohort of 187 patients in the original EACH study.
2. Differences in baseline characteristics, as summarized in Supplementary Table S5, indicated that our patients exhibited more high-risk features. These differences probably reflect the differences between the clinical trial conditions and real-world scenarios.

The prevalence of cirrhosis was notably higher (90.8%) in the present study compared with 55% in the EACH study. Cirrhosis is a pivotal factor affecting the survival of patients with HCC [20,21,22], with decompensated cirrhosis contributing to poorer prognosis, owing to both cirrhosis itself and decreased chemotherapy tolerability among these patients [23,24]. Furthermore, the rates of CTP B (26.3%) and ascites (10.5%) were higher in the present study compared with the EACH study (11.0% and 3.3%, respectively), further highlighting the increased risk of decompensated cirrhosis in our patient cohort.

With respect to tumor burden, the larger maximal tumor diameter (11.0 cm) in the present study contrasted with that of the EACH study (7.85 cm). It is well established that high tumor burden correlates with poor survival in patients with HCC [20,25,26,27]. Furthermore, elevated AFP levels indicate poorer prognosis [20,28,29]. AFP levels were notably higher in our study (5630.5 ng/dL) compared with those of the EACH study (1312 ng/dL).

In addition, 73.7% of the patients in our study exhibited HBV infection compared with 92.9% in the original EACH study. Furthermore, alcohol-related cirrhosis accounted for 15.8% of our patient population; however, no patient in the EACH study suffered from this condition. Notably, a comprehensive database study has reported that patients with HBV-associated HCC exhibit superior survival outcomes than those with other etiologies [30]. Moreover, alcohol-related cirrhosis has an increased mortality risk and cirrhosis decompensation compared with chronic HCV infection or NAFLD-related cirrhosis, coupled with a higher probability of acute-on-chronic liver failure and hepatic encephalopathy than HBV cirrhosis [30,31].

3. The AUROC and Harrel’s C-index metrics of our study model were 0.621 and 0.589, respectively. These values signified the considerably less effective discrimination performance when compared with the values of 0.7 for both metrics in the original EACH study [15]. Importantly, the variables incorporated into the proposed survival nomogram in our study did not exhibit statistically significant associations with survival, as previously observed in the EACH study. In our cohort, only TB displayed statistical significance. This discrepancy possibly contributed to the poor performance and discriminatory capability of the nomogram in our patient population.

Regarding the ALBI grade, although both the AUROC and Harrell’s C-index were 0.663, significant differences were observed in both numbers and statistical significance between each grade when using the Kaplan–Meier method and log-rank test. In the present study, we demonstrated that this tool can effectively differentiate patients receiving FOLFOX4 into three distinct prognostic categories, whether considering the entire cohort or the first-line cohort. Patients with ALBI grade 1 exhibited the longest median OS, followed by those with grades 2 and 3. This outcome affirms that the ALBI grade can independently predict survival in the context of chemotherapy, aligning with the results of previous studies on treatments such as sorafenib, lenvatinib, regorafenib, nivolumab, and pembrolizumab [19,32,33,34,35,36,37,38,39,40,41].

Although this study did not aim to directly compare these two prognostic tools, the differences in why the ALBI grade seemed to be effective in predicting the prognosis of patients with advanced HCC treated with FOLFOX4 while the survival nomogram did not could be explained by the different factors included in the model. In the survival nomogram, six variables were included, but only TB was found to be a significant factor affecting survival in this study. Therefore, it could be inferred that the high-risk group identified by the survival nomogram may not have actually been high risk because its score was influenced by other non-significant factors, resulting in the OS in the high-risk group from the nomogram being longer than the high-risk group (ALBI grade 3) identified by the ALBI grade (3.70 months vs. 1.58 months). Thus, it could be implied that the high risk group identified by ALBI (grade 3) was indeed high risk, as it was composed of two significant factors (albumin and TB) that affect survival. This helps explain why the number of patients classified as high risk appeared to differ between the two prognostic tools; there were 40 patients classified as high risk in the nomogram, while only 10 patients were identified as high risk (grade 3) by the ALBI grade.

While the ALBI grade may serve as a prognostic tool under chemotherapy conditions, it may not be the most optimal choice. In general, we expect ideal tools for risk classification to possess AUROC and Harrell’s C-index values of >0.7, signifying strong accuracy and agreement between the predicted and observed outcomes [42,43]. Nevertheless, our study is the first to use the ALBI grade in the context of the FOLFOX4 regimen. Additional insights into the effectiveness of the ALBI grade for practical use can be achieved by using a larger sample size and performing external validation.

With respect to effectiveness, we observed that PFS was consistent with the comparison studies; however, OS and ORR displayed lower values than those documented in previous studies [11] (Supplementary Table S6). This observation can be elucidated using the following factors. The diminished survival rates evident in our cohort can be attributed to differences in baseline characteristics inherent to a real-world environment, including factors such as decreased chemotherapy dosage intensity and subsequent treatment lines. Furthermore, the lower ORR observed in the ITT analysis may have been affected by an unexpectedly limited number of radiological assessments in our study, in which only 50% of the patients underwent CT for response evaluation.

To the best of our knowledge, our study is the first to externally validate the proposed survival nomogram and the ALBI grade in patients with advanced HCC who received the FOLFOX4 regimen. Our study highlights the effectiveness of the FOLFOX4 regimen in managing advanced HCC in a real-world setting, which often includes higher heterogeneity compared with the controlled environment of clinical trials.

However, our study has some limitations that should be carefully considered. First, it was conducted at a single center; therefore, the sample size was small. Second, the inherent retrospective nature of the study inevitably resulted in instances of missing data. Lastly, the incidence of radiological assessments was unexpectedly lower, potentially affecting actual ORR and PFS outcomes.

In summary, the ALBI grade is a potential prognostic tool for differentiating patients with advanced HCC who can achieve maximal benefits from the FOLFOX4 regimen. However, the proposed survival nomogram does not provide distinct discrimination. Nevertheless, it exhibited a tendency toward worse outcomes in high-risk patients. Therefore, in clinical settings, the FOLFOX4 regimen should be considered an option for patients with ALBI grade 1 who cannot receive immunotherapy or targeted therapy. Nevertheless, additional investigations are warranted to validate the survival nomogram and ALBI grade in a larger and prospective cohort.

## Figures and Tables

**Table 1:** Baseline characteristics.

	Total (*n* = 76)	First-Line Chemotherapy Cohort (*n* = 56)	Later-Line Chemotherapy Cohort (*n* = 20)
Setting, *n* (%)			
First-line treatment	56 (73.7)	56 (100)	-
Second-line treatment	15 (19.7)	-	15 (75.0)
Third-line treatment	3 (4.0)	-	3 (15.0)
Fourth-line treatment	2 (2.6)	-	2 (10.0)
Sex, *n* (%)			
Women	13 (17.1)	9 (16.1)	4 (20.0)
Men	63 (82.9)	47 (83.9)	16 (80.0)
Age, mean (SD), years	56.5 (9.8)	55.9 (10.1)	58.0 (8.9)
BMI, median (IQR), kg/m^2^	21.8 (19.4, 24.5)	22.2 (20.1, 24.6)	21.1 (19.2, 23.5)
Healthcare system, *n* (%)			
CSMBS	12 (15.8)	6 (10.7)	6 (30.0)
Social security	7 (9.2)	7 (12.5)	0 (0)
Universal coverage	53 (69.7)	42 (75.0)	11 (55.0)
Self-payment	4 (5.3)	1 (1.8)	3 (15.0)
ECOG, *n* (%)			
0	9 (11.8)	5 (8.9)	4 (20.0)
1	64 (84.2)	49 (87.5)	15 (75.0)
2	3 (3.9)	2 (3.6)	1 (5.0)
Cirrhosis, *n* (%)	69 (90.8)	52 (92.9)	17 (85.0)
CTP class, *n* (%)			
A	56 (73.7)	39 (69.7)	17 (85.0)
B	20 (26.3)	17 (30.3)	3 (15.0)
Etiology *, *n* (%)			
HBV	56 (73.7)	41 (73.2)	15 (75.0)
HCV	11 (14.5)	8 (14.3)	3 (15.0)
Alcohol	12 (15.8)	9 (16.1)	3 (15.0)
NAFLD	2 (2.6)	1 (1.8)	1 (5.0)
PSC	1 (1.3)	1 (1.8)	0 (0)
Number of liver tumors, *n* (%)			
0	8 (10.5)	4 (7.1)	4 (20.0)
1–5	35 (46.1)	28 (50.0)	7 (35.0)
6–10	4 (5.2)	2 (3.6)	2 (10.0)
>10	24 (31.6)	17 (30.4)	7 (35.0)
Infiltrative	5 (6.6)	5 (8.9)	0 (0)
Maximum tumor diameter, mean (SD), cm	11.0 (6)	11.0 (5.6)	10.9 (7.5)
Vascular involvement, *n* (%)	43 (56.6)	34 (60.7)	9 (45.0)
Ascites, *n* (%)	8 (10.5)	6 (10.7)	2 (10.0)
BCLC, *n* (%)			
B	9 (11.8)	6 (11.4)	3 (15.0)
C	67 (88.2)	50 (89.3)	17 (85.0)
Extrahepatic metastasis, *n* (%)			
1	28 (36.8)	21 (37.5)	7 (35.0)
2	10 (13.2)	6 (10.7)	4 (20.0)
3	1 (1.3)	0 (0)	1 (5.0)
4	1 (1.3)	0 (0)	1 (5.0)
Metastatic site, *n* (%)			
Lymph node	14 (18.4)	8 (14.3)	6 (30.0)
Lungs	19 (25.0)	10 (17.9)	9 (45.0)
Pleura	3 (3.9)	1 (1.8)	2 (10.0)
Peritoneum	11 (14.5)	8 (14.3)	3 (15.0)
Adrenal gland	3 (3.9)	2 (3.6)	1 (5.0)
Bone	3 (3.9)	2 (3.6)	1 (5.0)
Ovary	1 (1.3)	1 (1.8)	0 (0)
Pancreas	1 (1.3)	1 (1.8)	0 (0)
Laboratory results			
TB, median (IQR), mg/dL	1.2 (0.7, 1.8)	1.3 (0.7, 1.8)	1.0 (0.5, 1.6)
AST, median (IQR), U/L	101.5 (61.5, 214.5)	117 (65.0, 280.5)	94.0 (54.5, 112)
ALT, median (IQR), U/L	45 (30.0, 78.8)	49 (29.8, 85.2)	40.5 (30.8, 70.0)
ALP, median (IQR), U/L	235 (133.0, 395.0)	263.5 (144.5, 398.5)	196.5 (95.5, 293.8)
Albumin, mean (SD), g/dL	3.5 (0.5)	3.4 (0.5)	3.6 (0.5)
Platelet count, median (IQR), /µL	201,500 (127,750, 261,000)	201,500 (121,250, 256,500)	205,000 (141,750, 262,500)
INR level, median (IQR)	1.2 (1.1, 1.3)	1.2 (1.2, 1.4)	1.2 (1.1, 1.2)
Creatinine, median (IQR), mg/dL	0.8 (0.7, 1.0)	0.8 (0.7, 1.0)	0.8 (0.7, 0.9)
AFP, median (IQR), ng/dL	5630.5 (152.1, 30,045)	5048.0 (135.2, 31,011)	11,231.5 (155, 30,045)
Previous treatment, *n* (%)			
Resection	13 (17.1)	5 (8.9)	8 (40.0)
TACE	31 (40.8)	19 (33.9)	12 (60.0)
RFA	7 (9.2)	6 (10.7)	1 (5.0)
SBRT	2 (2.6)	2 (3.6)	0 (0)
Doxorubicin	4 (5.3)	0 (0)	4 (20.0)
Sorafenib	12 (15.8)	0 (0)	12 (60.0)
Regorafenib	2 (2.6)	0 (0)	2 (10.0)
Nivolumab	3 (3.9)	0 (0)	3 (15.0)
Atezolizumab plus bevacizumab	1 (1.3)	0 (0)	1 (5.0)
Durvalumab plus tremelimumab	2 (2.6)	0 (0)	2 (10.0)

SD, standard deviation; IQR, interquartile range; BMI, body mass index; CSMBS, civil servant medical benefit scheme; ECOG, Eastern Cooperative Oncology Group; CTP, Child–Turcotte–Pugh; HBV, hepatitis B virus; HCV, hepatitis C virus; NAFLD, nonalcoholic fatty liver disease; PSC, primary sclerosing cholangitis; BCLC, Barcelona Clinic Liver Cancer; TB, total bilirubin; AST, aspartate aminotransferase; ALT, alanine aminotransferase; ALP, alkaline phosphatase; INR, international normalized ratio; AFP, alpha-fetoprotein; TACE, trans-arterial chemoembolization; RFA, radiofrequency ablation; and SBRT, stereotactic body radiotherapy. * Each patient may have >1 etiologies.

**Table 2:** Response rate.

	Total (*n* = 76)	First-Line Chemotherapy Cohort (*n* = 56)	Later-Line Chemotherapy Cohort (*n* = 20)
Evaluable, *n* (%)	40 (52.6)	26 (46.4)	14 (70.0)
Complete response, *n* (%)	(0)	0 (0)	0 (0)
Partial response, *n* (%)	9 (11.8)	5 (8.9)	4 (20.0)
Stable disease, *n* (%)	15 (19.7)	10 (17.9)	5 (25.0)
Progressive disease, *n* (%)	16 (21.1)	11 (19.6)	5 (25.0)
ORR as per ITT, *n* (%)	9 (11.8)	5 (8.9)	4 (20.0)
ORR as per assessable, *n* (%)	9 (22.5)	5 (19.2)	4 (23.5)
DCR as per ITT, *n* (%)	24 (31.5)	15 (26.8)	9 (45.0)
DCR as per assessable, *n* (%)	24 (60.0)	15 (57.7)	9 (64.3)

ORR, objective response rate; DCR, disease control rate; and ITT, intention to treat.

**Table 3:** Overall survival evaluated using the survival nomogram.

Median OS, Months (95% CI)	Survival Nomogram			*p*-Value
	Low Risk	Intermediate Risk	High Risk	
Entire cohort (*n* = 76)	9.82 (5.32, NA)	10.64 (3.75, NA)	3.70 (2.69, 9.26)	0.23
First-line cohort (*n* = 56)	5.32 (3.48, NA)	8.89 (3.75, NA)	3.29 (2.43, NA)	0.62
Later-line cohort (*n* = 20)	12.43 (9.82, NA)	13.27 (2.66, NA)	4.73 (2.69, NA)	0.50

OS, overall survival; CI, confidence interval.

**Table 4:** Overall survival evaluated using the ALBI grade.

Median OS, Months (95% CI)	ALBI Grade			*p*-Value
	Grade 1	Grade 2	Grade 3	
Entire cohort (*n* = 76)	9.82 (7.13, NA)	6.83 (3.16, 15.20)	1.58 (0.85, NA)	0.00024
First-line cohort (*n* = 56)	9.63 (4.11, NA)	5.26 (3.15, 15.20)	1.05 (0.85, NA)	0.0025
Later-line cohort (*n* = 20)	13.99 (9.82, NA)	10.87 (2.73, NA)	2.69 (NA, NA)	0.14

OS, overall survival; CI, confidence interval.

## Data Availability

The datasets used and/or analyzed during the current study are available from the corresponding author on reasonable request.

## Supplementary Materials

The following supporting information can be downloaded at: https://www.mdpi.com/article/10.3390/jpm14040403/s1, Figure S1: The proposed survival nomogram; Figure S2: Formula used to calculate the ALBI grade and its interpretation; Figure S3: Overall survival of the patients undergoing different treatment lines; Figure S4: Progression-free survival of the patients undergoing different treatment lines; Figure S5: Overall survival of the risk groups of the first-line cohort classified based on the proposed survival nomogram; Figure S6: Overall survival of the patients in the first-line cohort classified using the ALBI grade; Table S1: Categorization of the patients into three risk groups and three grades using the survival nomogram and ALBI grade; Table S2: Treatment information and subsequent treatments; Table S3: Multivariate Cox proportional hazards regression model for the survival nomogram; Table S4: Multivariate Cox proportional hazards regression model for the ALBI grade; Table S5: Differences in the baseline characteristics between the patients in our study and those in the EACH study; Table S6: Differences in the efficacy of the FOLFOX or XELOX chemotherapy regimens in clinical trials enrolling patients with advanced hepatocellular carcinoma.
