# Supplementary material for: The Performance of a Survival Nomogram and Albumin–Bilirubin Grade as Prognostic Tools in Advanced Hepatocellular Carcinoma Treated with FOLFOX4
Source: J Pers Med. 2024 Apr 11;14(4):403. doi: 10.3390/jpm14040403 (PMC11051573; doi:10.3390/jpm14040403)
Supplement: Supplementary file 1 [file jpm-14-00403-s001.zip › jpm-2946229-supplementary.pdf]

Supplementary Figure S1 The proposed survival nomogram

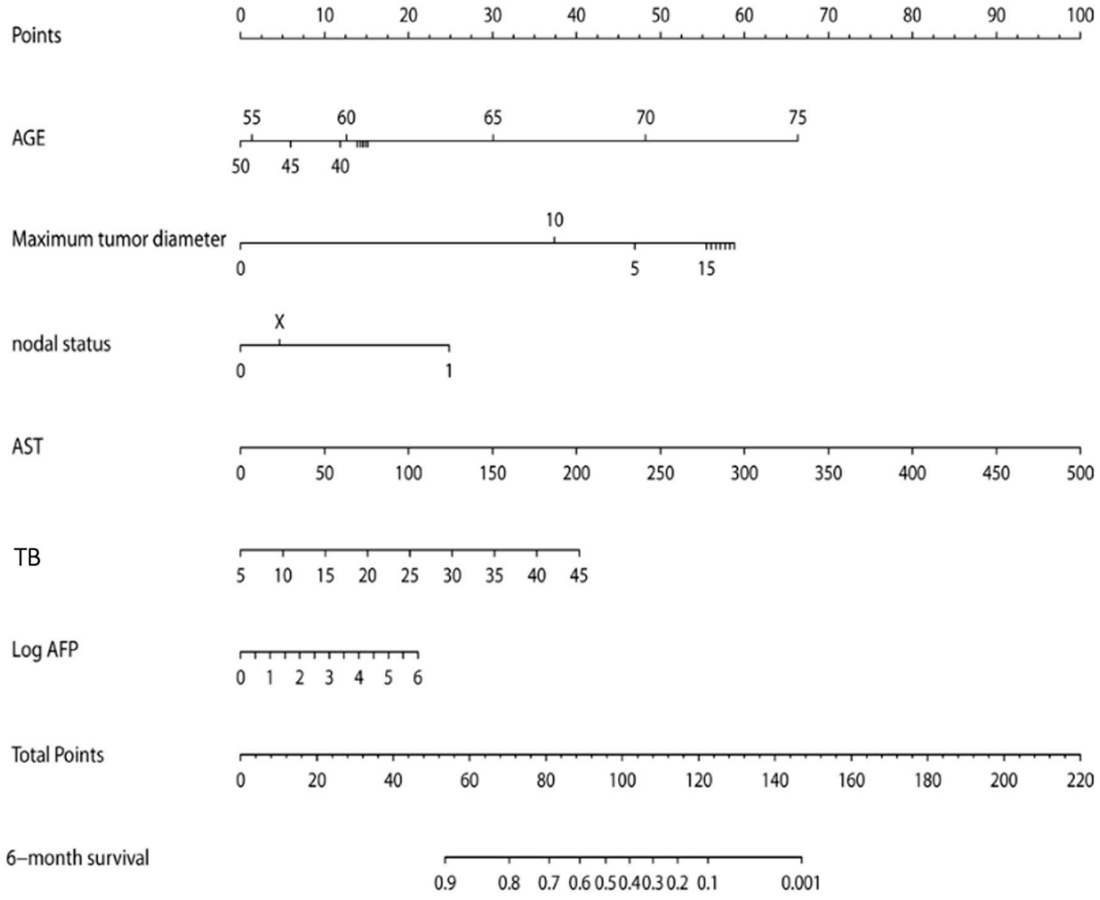

AST, aspartate aminotransferase; TB, total bilirubin; and AFP, alpha-fetoprotein.

Supplementary Figure S2 Formula used to calculate the ALBI grade and its interpretation

FORMULA

$$ALBI = (\log_{10} \text{bilirubin} \times 0.66) + (\text{albumin} \times -0.085)$$

- Bilirubin is in μmol/L and albumin is in g/L

INTERPRETATION

ALBI score	ALBI grade
≤ -2.60	1
> -2.60 to ≤ -1.39	2
> -1.39	3

Supplementary Figure S3 Overall survival of the patients undergoing different treatment lines

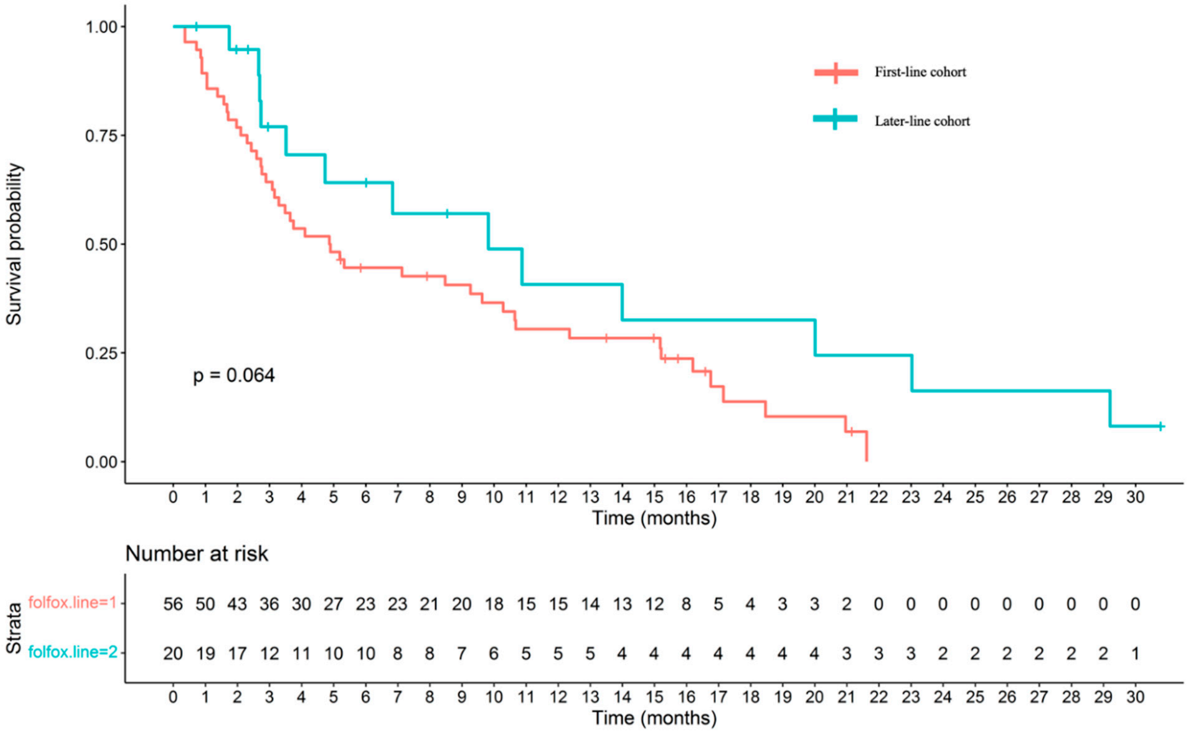

Supplementary Figure S4 Progression-free survival of the patients undergoing different treatment lines

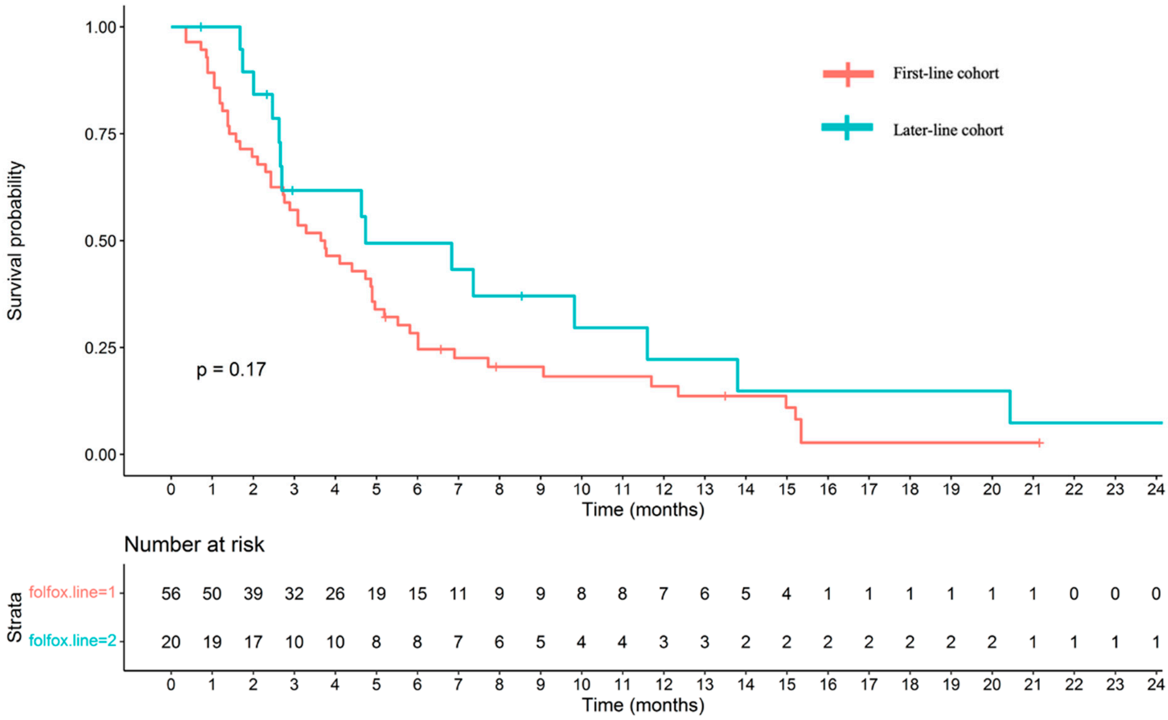

Supplementary Figure S5 Overall survival of the risk groups of the first-line cohort classified based on the proposed survival nomogram

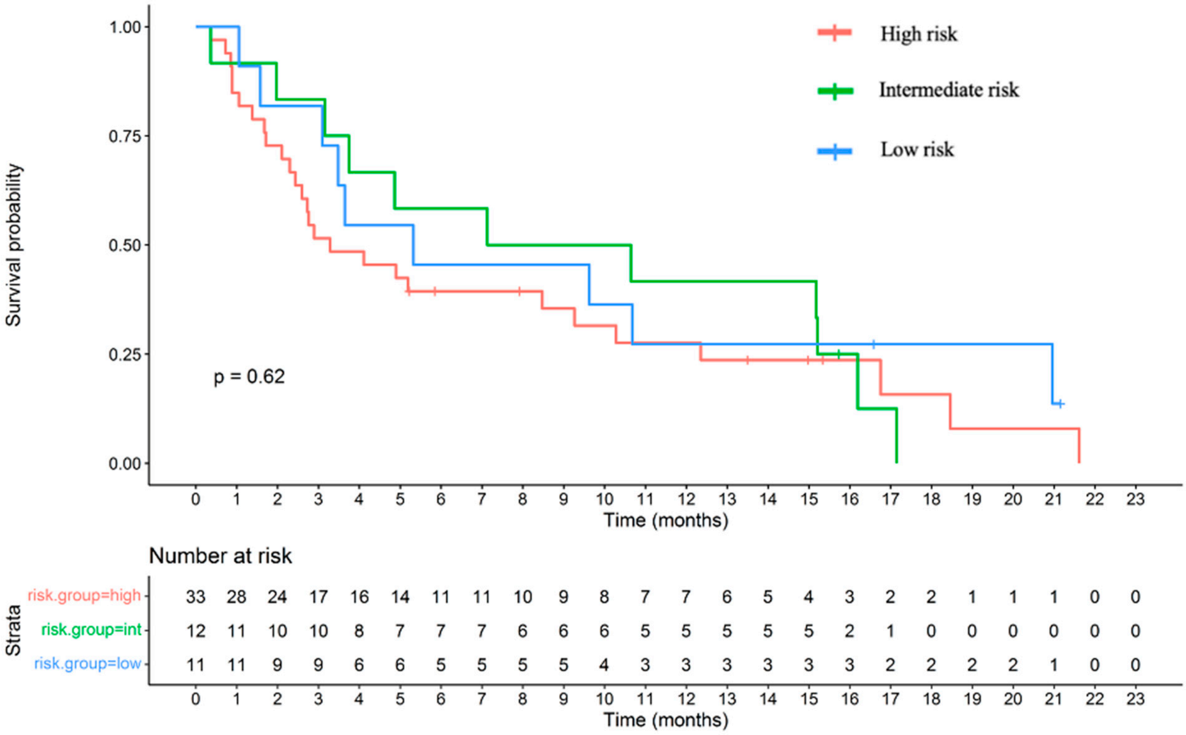

Supplementary Figure S6 Overall survival of the patients in the first-line cohort classified using the ALBI grade

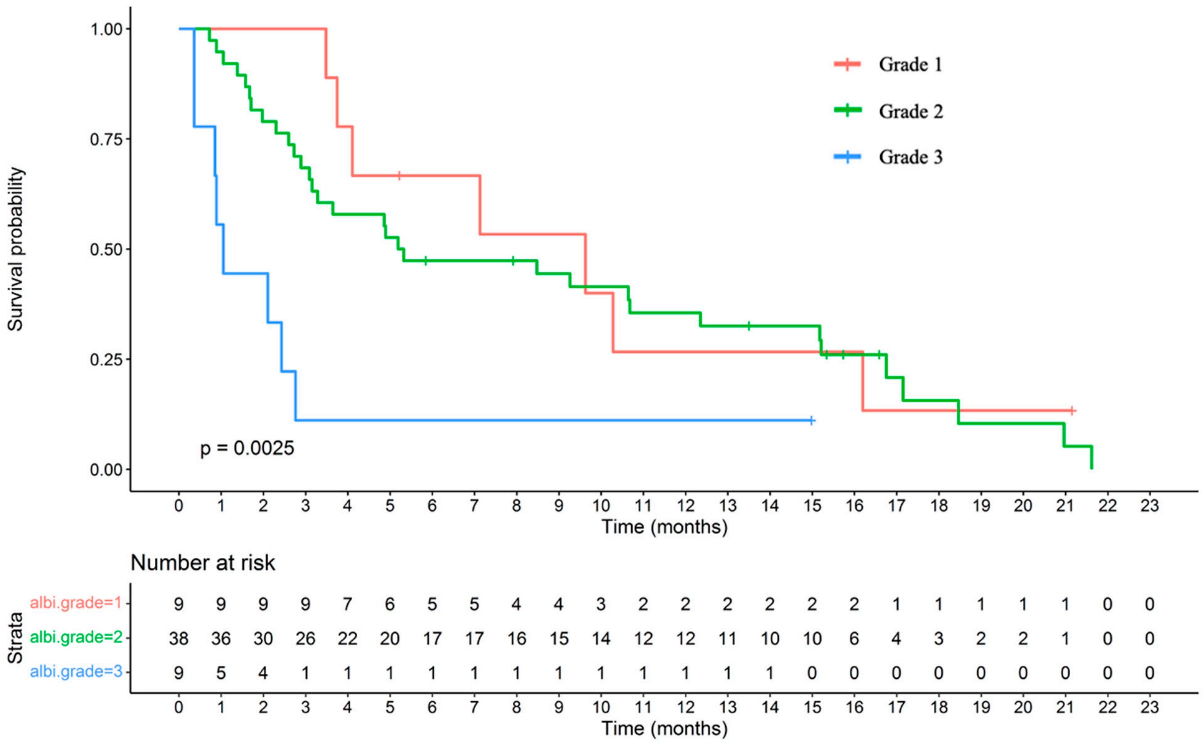

Supplementary Table S1 Categorization of the patients into three risk groups and three grades using the survival nomogram and ALBI grade

Number of patients, n (%)	Survival nomogram			ALBI grade		
	Low-risk	Intermediate-risk	High-risk	1	2	3
Total	17 (22.4)	19 (25.0)	40 (52.6)	15 (19.7)	51 (67.1)	10 (13.2)
First-line cohort	11 (19.6)	12 (21.4)	33 (59.0)	9 (16.1)	38 (67.9)	9 (16.1)
Later-line cohort	6 (30.0)	7 (35.0)	7 (35.0)	6 (30.0)	13 (65.0)	1 (5.0)

Supplementary Table S2 Treatment information and subsequent treatments

	Total (n = 76)	First-line chemotherapy (n = 56)	Later- line chemotherapy (n = 20)
**Number of FOLFOX4 cycles**, median (IQR)	3 (2.0, 8.0)	2 (1.0, 7.2)	5 (2.8, 11.2)
**Oxaliplatin**			
Starting dose, n (%)			
- Full dose	40 (52.6)	28 (50.0)	12 (60.0)
- First-level reduction	31 (40.8)	23 (41.1)	8 (40.0)
- Second-level reduction	5 (6.6)	5 (8.9)	0 (0)
Dose reduction, n (%)	58 (76.3)	42 (75.0)	16 (80.0)
**5-FU**			
Starting dose, n (%)			
- Full dose	67 (88.2)	50 (89.3)	17 (85.0)
- First-level reduction	7 (9.2)	4 (7.1)	3 (15.0)
- Second-level reduction	2 (2.6)	2 (3.6)	0 (0)
Dose reduction, n (%)	20 (26.3)	12 (21.4)	8 (40.0)
**Discontinuation of FOLFOX4**, n (%)			
Complete course	3 (3.9)	2 (3.6)	1 (5.0)
Death	9 (11.8)	8 (14.3)	1 (5.0)
Liver decompensation	13 (17.1)	10 (17.9)	3 (15.0)
Patient preference	3 (3.9)	3 (5.4)	0 (0)
Progressive disease	30 (39.5)	21 (37.5)	9 (45.0)
Poor performance status (declined)	9 (11.8)	8 (14.3)	1 (5.0)
Referred to nearby hospital	1 (1.3)	1 (1.8)	0 (0)
Toxicity	1 (1.3)	0 (0)	1 (5.0)
**Subsequent therapy**, n(%)	12 (15.8)	8 (14.3)	4 (20.0)
Systemic therapy, n(%)	9 (11.8)	7 (12.5)	2 (10.0)
- Doxorubicin	6 (7.9)	5 (8.9)	1 (5.0)
- FOLFOX4 (beyond progression)	3 (3.9)	2 (3.6)	1 (5.0)
Palliative radiotherapy, n(%)	3 (3.9)	1 (1.8)	2 (10.0)

IQR, interquartile range; FOLFOX, folinic acid (leucovorin), 5-fluorouracil (5-FU), and oxaliplatin.

Supplementary Table S3 Multivariate Cox proportional hazards regression model for the survival nomogram

**Variable**	**Hazard ratio (95% CI)**	**P-value**
Age	0.98 (0.96, 1.00)	0.075
Lymph node	1.21 (0.57, 2.58)	0.613
TB	1.27 (1.12, 1.43)	<0.001
AST	1.002 (1.00, 1.004)	0.052
Maximum tumor diameter	0.99 (0.95, 1.04)	0.731
Log AFP	1.06 (0.96, 1.16)	0.263

AST, aspartate aminotransferase; AFP, alpha-fetoprotein; CI, confidence interval; and TB, total bilirubin.

Supplementary Table S4 Multivariate Cox proportional hazards regression model for the ALBI grade

**Variable**	**Hazard ratio (95% CI)**	**P-value**
Albumin	0.67 (0.37, 1.24)	0.206
Total bilirubin	1.27 (1.14, 1.43)	<0.001

CI, confidence interval.

Supplementary Table S5 Differences in the baseline characteristics between the patients in our study and those in the EACH study

	**Our study (n = 76)**	**EACH study (n = 187)**
Age (mean), years	56.5	50
Men, %	82.9	90.2
Cirrhosis, %	90.8	55.4
Child–Turcotte–Pugh score, %		
A	73.7	88.6
B	26.3	11.4
Etiology, %		
HBV	73.7	92.9
HCV	14.5	4.97
Alcohol	15.8	-
Number of liver tumors, %		
0	10.5	3 (1–11)
1–5	46.1	Median (IQR)
6–10	5.2	
>10	31.6	
Infiltrative type	6.6	
Maximum tumor size, cm (SD)	11.0 (6)	7.85 (4.75–11.7)<br>Median (IQR)
AFP, ng/dL	5630.5	1312
BCLC, %		
B	11.8	21.2
C	88.2	78.8
Extrahepatic metastasis, %	52.6	56.5
Portal vein thrombosis, %	56.6	60.9
Ascites, %	10.5	3.3
TB, mg/dL	1.2	0.91
AST, U/L	101.5	38

HBV, hepatitis B virus; HCV, hepatitis C virus; SD, standard deviation; IQR, interquartile range; AFP, alpha-fetoprotein; BCLC, Barcelona Clinic Liver Cancer; ALBI score, albumin–bilirubin score; TB, total bilirubin; and AST, aspartate aminotransferase.

Supplement Table S6 Differences in the efficacy of the FOLFOX or XELOX chemotherapy regimens in clinical trials enrolling patients with advanced hepatocellular carcinoma

Study	Design	No. of patients	Treatment	DCR (%)	ORR (%)	TTP/PFS	Median OS	Cirrhosis (%)	CTP A/B (%)	2L setting (%)
**Qin S et al.**	Open-label, randomized, phase III	184/187	FOLFOX4/doxorubicin	52.17	8.15	2.93 months	6.4 months	55.7	88.6/11.4	20.6
**Qin S et al.**	Open-label, randomized, phase III	140/139	FOLFOX4/doxorubicin	47.1	8.6	2.4 months	5.7 months	55.4	90/10	20.7
**Yang L et al.**	Single-arm	77	FOLFOX4	55.6	4.2	2.7 months	6.1 months	-	77.9/22.1	-
**Zhou et al.**	Single-arm	20	FOLFOX	60.0	20.0	2.2 months	5.0 months	-	68.8/31.2	-
**Yang L et al.**	Clinical observation	31	CAPOX	42.9	7.1	2.9 months	N/A	54.8	80.6/19.4	-
**Yin Z et al.**	Clinical observation	20/20	CAPOX/FOLFOX6	55.0	5.0	2.1 months	9 months	-	-	-
**He SL et al.**	Single-arm	32	CAPOX	62.5	21.9	4.2 months	9.2 months	-	-	-
**Wang F et al.**	Clinical observation	13	FOLFOX4/CAPOX	61.5	15.4	3.9 months	8.0 months	-	-	100.0 after sorafenib
**Our study**	Retrospective	76	FOLFOX4	31.5 (ITT) 60.0 (assessable)	11.8 (ITT) 22.5 (assessable)	4.11 months	5.32 months	90.8	73.7/26.3	26.3

FOLFOX, folinic acid (leucovorin), 5-fluorouracil, and oxaliplatin; CAPOX, capecitabine and oxaliplatin; DCR, disease control rate; ORR, objective response rate; TTP, time to progression; PFS, progression-free survival; OS, overall survival; CTP, Child–Turcotte–Pugh; ITT, and intention to treat.
